# The human-robot interaction scale database

**DOI:** 10.3389/frobt.2026.1846413

**Published:** 2026-07-28

**Authors:** Laura Saad, Eileen Roesler, Elizabeth Phillips, J. Gregory Trafton

**Affiliations:** 1 National Research Council Postdoctoral Research Associate, Washington, DC, United States; 2 Navy Center for Applied Research in Artificial Intelligence, U.S. Naval Research Laboratory, Washington, DC, United States; 3 Department of Psychology, George Mason University, Fairfax, VA, United States

**Keywords:** human-robot interaction, psychometrics, questionnaires, reliability, scales, subjective measures, validity

## 1 Introduction

Measuring the human in human-robot interaction (HRI) contexts is critically important if we want the robots we design to meet the high expectations we have for them: to be useful, helpful, reliable, trustworthy, transparent, and so on. The human perspective within HRI can be measured with objective and subjective measures. Objective measures, such as task completion time or task performance, can provide valuable evidence regarding the quality of a robot system, interface, or interaction (Zimmerman et al., 2022; Apraiz et al., 2023; Hopko et al., 2022; Daniel et al., 2013). However, when a human interacts with a robot, it is also important to account for and measure their internal psychological experiences, such as their perceptions, emotions, and other latent processes (Corneille and Gawronski, 2024; Norman, 2007; Parasuraman and Riley, 1997; Picard, 1999). These experiences are not directly observable and therefore cannot be captured through behavioral observation or objective metrics alone (Haney and Liang, 2024). Subjective measures, such as scales, play a critical role in accessing these latent, unobservable constructs. Constructs are defined as underlying psychological characteristics or processes that are not directly measurable (DeVellis and Thorpe, 2021). Subjective measures provide valuable insight into a person’s attitudes, thoughts, feelings, expectations, and perceptions, offering a window into the individual’s internal state that would otherwise remain hidden. As these constructs are inherently unobservable, subjective measures such as scales are necessary for gaining a complete understanding of the human perspective during HRI.

In HRI research, scales are frequently used to capture the human perspective and play a crucial role in contributing to advancements in robotics and related technologies. Evidence for their widespread use comes from Zimmerman et al. (2022), which detailed a comprehensive analysis of self-report measures in proceedings from the Human-Robot Interaction (HRI) and Robot and Human Interactive Communication (RO-MAN) conferences (two of the primary publication venues for HRI research) over a 7 year period (2015–2021). A major takeaway from this investigation was that the majority of publications from these conferences (889/1,464, or 61%) incorporated scales as dependent measures in study designs. However, of particular importance to this paper, it was also revealed that the vast majority of those that included scales (815/889, or 92%) relied on custom scales to capture the constructs of interest. The term “custom” refers to the customization of either an existing scale or the creation of a scale that has been customized for a specific purpose (e.g., a researcher comes up with a handful of items they believe will capture their construct of interest); in either case, they are called “custom” scales because they have not been validated. Therefore, a straightforward conclusion from these results is that while scales clearly play a central role in HRI research, the widespread usage of non-validated scales raises concerns about the reliability and replicability of existing findings, as such measures are often not comparable across studies and have not undergone rigorous scale development and testing.

More recently, Schrum et al. (2023) investigated HRI conference proceedings from 2016 to 2020 (excluding alt.HRI and Late-Breaking Reports) that specifically reported using Likert scales. The authors reported that a majority of these scales (72.5%) were not appropriately developed or validated according to acceptable psychometric standards. Importantly, an additional 9.8% of scales used were custom scales. One potential reason researchers use custom scales is that they may be unaware of existing scales that meet their research goals. Since HRI is inherently multidisciplinary, relevant scales can originate from many different fields, such as robotics, psychology, sociology, human factors, human-computer interaction, ergonomics, or elsewhere. Taken together, this evidence for limited use of validated scales is concerning and underscores a critical issue in the field: the scale selection process in HRI research can and should be improved.

Measurement is a fundamental component of science. Measurements give us access to information that we cannot intuit on our own. In HRI contexts, the information we gain from subjective and objective measures are used to better understand how humans complete tasks, how they perceive other entities, and the quality of the performance, among other things. This information is then used to inform and develop better theories and experiments and design better software and hardware which in turn, advances the field forward. Therefore, the quality of the measure (i.e., its reliability, or the extent to which it yields similar results under similar conditions, and its validity, or the degree to which it accurately measures what it is intended to measure) is crucially important. Without this, we run the risk of drawing conclusions and designing research based off of results that are not replicable or worse, come from measures that do not actually measure the intended construct.

For a developing field like HRI, it is imperative that foundational results be based on reliable measures, as the community has the potential to develop groundbreaking theories and applications with far-reaching impact. However, many aspects of the HRI research community that contribute positively to the impact of the work, such as the interdisciplinary quality of the community, appear to leave it susceptible to measurement pitfalls. The recent evidence (Schrum et al., 2023; Schrum et al., 2020; Zimmerman et al., 2022) that the field relies primarily on non-validated or custom scales is problematic for a number of reasons. First, as previously stated, customized scales do not guarantee reliable or valid results. Additionally, customizing previously validated scales makes the integration of findings across studies more difficult, since the customized scale is typically only used in a very small number of studies and, in some cases, only once. This then limits the potential for incremental knowledge gains. More broadly, the use of improperly developed scales can lead to inappropriate conclusions and wasted resources which should be avoided whenever possible.

While all of these points are important to consider and may leave the interested researcher rightfully wary of customizing scales, it is also true that developing psychometrically valid scales is a significant undertaking. The process is not always straightforward, especially for those without training in psychometric theory (which is the case for many HRI researchers). Developing scales can also be time and resource consuming for research teams. Additionally, finding and critically evaluating existing scales can be an even more difficult task. Many constructs have multiple scales (e.g., trust has at least seven different scales used in HRI research (Charalambous et al., 2016; Yagoda and Gillan, 2012; Malle and Ullman, 2021; Malle and Ullman, 2023; Körber, 2019; Jian et al., 2000; Schaefer, 2016)) making it difficult to determine which scale is appropriate to use in which contexts. For HRI researchers, finding appropriate scales can be a challenge. Yet for the field as a whole, progress is impossible without reliable and valid measurement tools.

Here, we propose a solution to these challenges. We have developed a database (https://hriscaledatabase.psychology.gmu.edu/) which acts as the first publicly available, centralized, online repository of scales in HRI. The database includes more than 50 of the most commonly used scales in HRI. Additionally, all the scales have been evaluated by experts and rated according to a previously published psychometric guideline (Saad et al., 2025). Each scale has its own webpage which contains all of the relevant information a researcher might need to know when deciding whether or not to use a scale in their research. These critical evaluations, represented as numeric guideline ratings, provide researchers with the tools to efficiently compare scales that measure a specific construct of interest. Importantly, the guideline ratings provide additional information about the quality of these scales (or at least the quality of reporting on how the scales were developed), which can then be used by future researchers to make more informed decisions and, ideally, include higher-quality scales in their work.

The database has significant potential to positively impact HRI research. First, gathering scales to a centralized location will improve the efficiency of research by reducing the resources required to search for scales, which can then be devoted to other aspects of the research process. Second, having a consistent way to critically evaluate scales (i.e., the guideline (Saad et al., 2025)) will also make it easier to assess the psychometric quality of a scale. This standardization in scale evaluation will also make the comparison across scales within a specific construct easier, thereby further simplifying the scale selection process for future researchers^1^.

Additionally, the guideline can also serve as a benchmark for scale developers, offering a standard to strive toward in achieving high ratings. This will in turn improve the quality and standard of future scales for the HRI community. Third, the database will also increase the level of standardization in the administration of scales. Each scale page contains the final items for that respective scale as well as the response scale lengths (e.g., a 5-point response scale versus a 7-point response scale) and endpoints labels (e.g., “Not at all” or “Very much”) for each of the items in the scale. As it is important to administer a previously validated scale in the format it was validated in (Schrum et al., 2023; DeVellis and Thorpe, 2021; Boateng et al., 2018; Clark and Watson, 2016), having easy access to this information should reduce the potential for misinterpretations or perpetuating mistakes in the literature. This will improve the replicability of research in this domain and make the comparison of results across publications more straightforward. Lastly, prioritizing the use of psychometrically valid scales will ensure that the conclusions we draw from responses to these scales are as valid and reliable as possible. As a result, the field can avoid drawing faulty conclusions from poorly developed scales which will lead to more replicable and interpretable research that can advance our knowledge in the field of HRI research.

Our aim is to provide an easy-to-use tool that can enable HRI researchers to efficiently and confidently find the current best scale to use for their research, or to compare scales to one another. The goal for this database is to enhance the quality of HRI scales to ensure our work remains rigorous and continues to have a positive impact on society.

## 2 Methods

The objective in collecting scales for the database was to include the most highly cited and commonly used scales in HRI research. Citation counts were used as a relative indicator of a scale’s prominence and use within the HRI community rather than as a strict inclusion criterion, and no minimum citation threshold was imposed. The goal of the selection process was therefore to provide broad coverage of commonly used and well-established HRI measures rather than to conduct an exhaustive systematic review. Additionally, scales did not need to be developed specifically for use in HRI research, but needed to be used by HRI researchers.

To populate the database, we started with widely used scales that were specific to the HRI community (e.g., Robotic Social Attributes Scale (RoSAS) (Carpinella et al., 2017), Godspeed (Bartneck et al., 2009), Negative attitudes towards robots (NARS) (Nomura et al., 2006b), and Multi-dimensional measure of trust (MDMT) (Malle and Ullman, 2021). Once those scales were identified, the subsequent process was iterative, involving continued literature reviews to refine and expand the set of commonly used and well-established scales, and was further informed by recommendations from researchers in the HRI community. Additional searches were conducted to identify scales that measured constructs relevant to the HRI community but that had not been recommended directly. These searches were conducted in the ACM/IEEE Proceedings of the International Conference on Human-Robot Interaction, the ACM Transactions on Human-Robot interaction journal, and Google Scholar. We tried to make sure that every construct had multiple scales representing them. This was in an effort to provide researchers with multiple options within a given construct and to expose them to varying definitions and measures, thereby supporting more informed decision-making when selecting scales for their research.

After identifying the publications in which the scale was originally developed and/or validated, the scales within each paper were rated using a guideline that was previously developed using best practices from the psychometrics scientific community (Saad et al., 2025) (see Table 1)^2^. The guideline was developed specifically for HRI researchers to provide a systematic method for evaluating scales and contains 13 questions that can be used to determine whether a scale was appropriately developed and validated. The guideline questions are grouped into three stages of the scale development and validation process: item development, scale development, and scale evaluation. The questions in the item development stage aim to ensure that the construct was clearly defined and that the scale items were developed according to commonly accepted procedures and also match with the construct definition as the authors defined it. Guideline questions in the scale development stage pertain to the process by which the authors actually developed and initially tested the scale. This includes an assessment of critical parts of this process such as determining adequate sample sizes and appropriate methods for determining scale factors and the item to factor relationship. Finally, guideline questions in the scale evaluation stage pertain to the assessment of the developed scale and includes information on how to look for and interpret reliability and validity assessments reported in a scale development paper.

**TABLE 1:** Guideline – choosing the “perfect” scale (Saad et al., 2025).

Stage	Question	Check
Item development	1. Is the construct clearly defined?	□
	2. Is the item generation process discussed (e.g., via a literature review, the delphi method, or crowd-sourcing)?	□
	3. Do the final items capture the construct as defined in the paper?	□
Scale development	4. Full initial set of items reported?	□
	5. Does the sample size meet the 10:1 participant-to-item criterion?	□
	6. Did developers use EFA, PCA, or rasch to determine item–factor relationships?	□
	7. Factor extraction method discussed?	□
	8. Factor loadings or item fits provided for all items?	□
	9. Is the item removal process described?	□
	10. Final item list reported?	□
Scale evaluation	11. Test for factor structure reported?	□
	12. Reliability reported (e.g., α , ω )?	□
	13. Validity tested (e.g., convergent, discriminant)?	□

The database applies these guidelines to scales that measure frequently used constructs in HRI. These evaluations can then help researchers determine whether the scales have been adequately developed and validated. For each guideline question, scales can receive a rating score of 1 (“yes the paper reported that the criteria for scale development was met”) or 0 (“no the paper did not report that the criteria was met”). The maximum number of points a scale can receive is 13/13, which is converted into a percentage (out of 100) in the database.

Higher scores on the guideline evaluation indicate that the paper being assessed has demonstrated a more effective and well-executed scale development and validation process, making comparisons across scales relatively straightforward for the researcher. However, we do not recommend that researchers choose scales based solely off of the guideline score. This is because the highest rated scale within a certain construct category may not actually be applicable for a given research purpose. A clear example can be found in the trust category, where the highest rated scale is the Trust in Industrial Human-Robot Collaboration scale (Charalambous et al., 2016). In their paper, the authors defined the construct of trust specifically in the context of industrial human-robot collaboration and therefore the items were designed to measure this construct (e.g., “I knew the gripper would not drop the components”). Importantly, researchers studying trust in other HRI contexts (e.g., military, medical, etc.) would likely find this scale inappropriate or unsuitable for their research. Lastly, we also do not encourage the reader to discard a scale simply because it did not receive a perfect score on the guideline. In the case that a scale does not meet all of the criteria in the guideline, we recommend considering the reported scale development process as a whole (i.e., does the construct definition match with the needs of the project, does the scale appear to have been adequately developed and validated) and consider whether the scale can still meet your research needs.

The process of rating the scales was conducted as follows. For each scale, at least two individuals rated the scale independently using the guideline questions. All raters were trained by experts in HRI and psychometric theory to promote consistent and accurate assessment. These experts have extensive training and backgrounds in cognitive psychology, cognitive science, computer science, and human factors, and all scales were reviewed by at least one of these experts. We note that while most guideline criteria were applied consistently across raters, some items, particularly those assessing the degree to which scale items captured the construct as defined by the original authors. In cases of disagreement, discrepancies were resolved through structured discussion of the guideline criteria until consensus was reached, with the goal of establishing a shared interpretation of the criteria rather than favoring any single rater’s judgment.

To evaluate the reliability of this rating procedure, interrater reliability analyses were conducted on a subset comprising 30% of the scales included in the database. Across these scales, raters demonstrated moderate agreement, with a mean Cohen’s κ of 0.72 (SD = 0.07), alongside high overall agreement rates (M = 84.95%, SD = 11.03%). Disagreements most frequently arose for guideline items involving construct interpretation. For example, the sociability subdimension of the HRIES scale defines sociability as “the social constructs that are positively related to the intent of interaction with others [40, 41, 134]. Sociability is the ability of an individual or a group of individuals to evolve in society” (Spatola et al., 2021, p. 1522), whereas the corresponding items assess attributes such as warmth, likeability, trustworthiness, and friendliness. Under a strict interpretation, these items may only partially reflect the stated construct definition; however, raters judged that the items adequately captured the positive social component of the construct. This example illustrates the types of interpretive decisions that occasionally contributed to disagreement and highlights the challenges inherent in evaluating broad or multifaceted construct definitions.

## 3 Database organization and content

The stated aim of the database is to serve as a useful tool for HRI researchers. To achieve this goal, the database is organized in a way that simplifies the process of finding the right scale for a specific project or researcher. Specifically, the database can be searched by construct label, such as “trust” or “safety”, or by general research area of interest to HRI, such as “social robotics”.

Within each construct label, the scales are listed in order of their score on the guideline. Each scale has its own page which includes critical information about the scale such as the construct definition as it has been reported in the paper including a direct quote of the definition, where possible. Additionally, each scale page also includes the rating (i.e., a percentage out of 100%) that the scale received based on the guideline as well as a table displaying specific information about where the scale gained or lost points according to the guideline criteria. There is also a comment section for each scale that contains additional clarification regarding specific guideline rating decisions, where necessary. For example, if a scale development paper reported conducting a set of analyses but misinterpreted the results, the scale still earned a point on the guideline for conducting that analysis, but the misinterpretation is noted to ensure that researchers can make informed decisions about the scale. Each scale page also includes a list of the final version of the items in the scale as reported by the authors in the paper, as well as a link to the paper and a citation. The scale page also includes a downloadable PDF of the blank scale as well as scale administration and scoring instructions, when available. Lastly, if an aspect of the scale has been updated (e.g., shortened, expanded, validated), the scale page includes a link to the most recent version of the scale clearly marked at the top of the page. This additional information allows the database to account for the iterative and self-correcting nature of science and ensures that the database captures the complete lifespan of a scale, whenever possible. Relatedly, the database will be updated on a yearly basis to ensure that it remains current and continues to reflect new developments in the literature.

The database also includes information about the guideline (Saad et al., 2025). Specifically, the database includes additional information about each individual guideline question so that researchers can easily determine which part of the scale showed weakness. These weaknesses can be used by later researchers to improve existing scales. The database also includes a glossary of definitions of terms that are commonly used in psychometric theory as an additional reference.

The database currently contains 53 scales capturing 12 frequently used HRI constructs and research domains (the complete list of scales included in the database can be found in Table 2). These constructs and labels were selected to reflect domains that are commonly assessed in HRI research and represented across widely used existing scales, rather than to provide an exhaustive or definitive taxonomy of HRI measurement. Instead, the labels were intended to serve as practical organizational categories for navigating the database. Additionally, the database is intended to be expandable as additional constructs and scales are identified and incorporated. Each domain is annotated with labels that capture both specific constructs (e.g., safety/danger) and broader topics (e.g., social). Those labels are: anthropomorphism, attitudes/attributes, mental models, mind/agency perception, morality, perceived embodiment, safety/danger, social, trust, usability, user experience, and workload. The ratings for these scales range from 15% (2/13 points) to 100% (13/13 points). The total number of citations across all scales included in the database is over 73,000 (as of 4/2/26).

**TABLE 2:** All scales in the HRI scale database with citations.

Scale name	Citation
Individual differences in anthropomorphism questionnaire child form (IDAQ-CF)	Severson and Lemm (2016)
Scale of social robot anthropomorphism (SSRA)	Chi et al. (2025)
Anthropomorphism rasch	Ruijten et al. (2019)
Individual differences in anthropomorphism scale (IDAQ)	Waytz et al. (2010)
Perceived empathy (RoPE)	Charrier et al. (2019)
Anthropomorphic tendencies scale (ATS)	Chin et al. (2004)
General attitudes towards robots scale (GAToRS)	Koverola et al. (2022)
Robotic social attributes scale (RoSAS)	Carpinella et al. (2017)
Self-efficacy in HRI (SE-HRI)	Pütten and Bock (2018)
Attitudes towards robots measure (ARM)	Spatola et al. (2023)
Robot anxiety scale (RAS)	Nomura et al. (2006a)
Negative attitudes towards robots scale (NARS)	Nomura et al. (2006b)
Educational robot attitude scale (ERAS)	Sisman et al. (2019)
Human robot interaction stress scale (HRISS)	Babamiri et al. (2024)
Multidimensional robot attitude scale (MRAS)	Ninomiya et al. (2015)
Godspeed scale	Bartneck et al. (2009)
Mental models of robots	Kiesler and Goetz (2002)
Robot threat assessment (RoTA)	Matthews et al. (2019)
Perception of agency (PA) scale	Trafton et al. (2024)
Dimensions of mind perception (MMP35)	Malle (2019)
Weisman mind perception	Weisman et al. (2017)
Dimensions of mind perception	Gray et al. (2007)
Unidimensional mind perception	Tzelios et al. (2022)
Perceived moral patiency scale (PMP)	Banks and Bowman (2023)
Perceived moral agency scale (PMA)	Banks (2019)
Robot’s rights and responsibilities scale (RRR)	Mays et al. (2025)
Moral concern for robots scale (MCRS)	Nomura et al. (2019)
Avatar embodiment	Gonzalez-Franco and Peck (2018)
EmCorp scale	Hoffmann et al. (2018)
Avatar embodiment - validated	Peck and Gonzalez-Franco (2021)
Perceived danger (PD)	Molan et al. (2025)
Psychological safety	Kamide et al. (2012)
Connection-coordination rapport scale (CCR)	Lin et al. (2025)
Human-robot interaction evaluation scale (HRIES)	Spatola et al. (2021)
Machines as social entities (MASE)	Hong (2024)
Frankenstein syndrome questionnaire (FSQ)	Syrdal et al. (2013)
Partner modeling questionnaire (PMQ)	Doyle et al. (2025)
Uncanny valley effect	Ho and MacDorman (2017)
Partner modeling in speech interfaces	Doyle et al. (2021)
Collaborative fluency	Hoffman (2019)
Trust in industrial human-robot collaboration	Charalambous et al. (2016)
HRI trust scale	Yagoda and Gillan (2012)
Multidimensional measure of trust (MDMT)	Malle and Ullman (2021)
Trust in automation - Körber (TiA)	Körber (2019)
Trust in automation - Jian (TIA)	Jian et al. (2000)
Trust perception Scale-HRI	Schaefer (2016)
Multidimensional measure of trust (MDMT) V2	Malle and Ullman (2023)
System usability scale (SUS)	Brooke (1996)
Usefulness, satisfaction, and ease of use scale (USE)	Lund (2001)
User experience questionnaire (UEQ)	Laugwitz et al. (2008)
Subjective workload assessment technique (SWAT)	Reid and Nygren (1988)
NASA task load index (NASA-TLX)	Hart (1986)
Human robot interaction workload measure (HRI-WM)	Yagoda (2010)

## 4 Analysis

**FIGURE 1:**
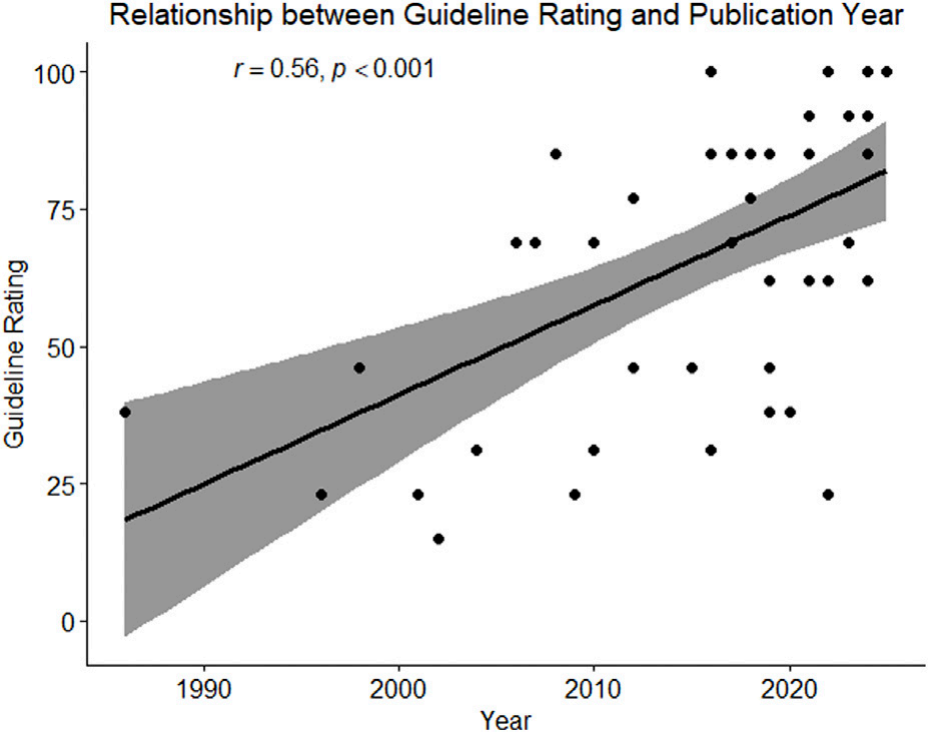
Relationship between year of publication and guideline (Saad et al., 2025) rating (95% CIs).

The database includes the most highly cited scales used in HRI research in the database. For example, the top three mostly highly cited scales in our database were the System Usability Scale (Brooke, 1996), the NASA-TLX workload scale (Hart, 1986), and the Godspeed Scale (Bartneck et al., 2009) which have 25,686, 22,181, and 4,279 citations (as of 4/2/26), respectively. Interestingly, those three scales have an average rating of 28%. One possible explanation for this is that these scales were developed based on dated methodologies (the average age of these three scales is 28 years old). As the guideline criteria are based on the latest advancements in scale development techniques, it may be the case that older scales have a lower rating simply because they did not have access to this information. Therefore, it follows that recently developed scales will have higher guideline scores because they have incorporated this updated information. Figure 1 plots the scale ratings of the scales included in the database over time. While the plot clearly suggests that scale quality is improving over time, we tested whether this relationship was statistically significant. To do this, we computed a Pearson’s correlation which indicated a large positive relationship between year of publication and guideline rating, *r* = 0.56, *p* < 0.001. These results indicate that the quality of HRI scales is unambiguously improving over time which in turn, suggest a bright outlook for the future of measurement in the field^3^. Importantly, these findings also suggest that the age of a scale may be a better indicator of its quality than its citation count. We therefore encourage HRI researchers to take this into consideration when selecting a scale^4^.

## 5 Using the database

The database contains a great deal of useful information about scales in HRI but its most useful feature is as a tool to help researchers find the “perfect” scale for their project. A perfect scale is one that claims to measure the construct of the researcher is interested in and was adequately developed and validated. To find their perfect scale, the researcher needs to first identify their construct of interest. This essentially amounts to defining the research question and dependent variables of interest. Once the construct of interest has been identified they should find the matching category label. They should then find the highest rated scale under that category label and read the corresponding construct definition and items to ensure it matches with their goals. If it does, then the search is over! The researcher can simply move on to the next step of their research project.

However, researchers may face a potential tradeoff in cases where the highest rated scale within a construct category may not actually be a perfect match to their needs. For example, researchers interested in measuring perceived anthropomorphism will find that the most highly rated scale in the database measures children’s tendencies to anthropomorphize (Severson and Lemm, 2016). This may make it inappropriate for use in their experiments if they are aimed at understanding perceived anthropomorphism in other populations. Additionally, it may also be the case that the scale using their preferred definition of the construct does not have a high rating on the guideline, indicating weaknesses in its measurement ability. It is up to the researcher to consider these tradeoffs when making the final decision about which scale to incorporate in their research. Lastly, it is important to note that the information provided in the database should be considered a starting point in the process of choosing the current best scale. We highly recommend that the researcher read the scale development paper for the scale they are interested in using and, importantly, also read the items in the scale and answer them in the context of the experiment themselves. A link to the source publication is provided on the webpage for every scale in the database and the items for each scale are listed directly in the database as well.

Though the database can help interested HRI researchers find the current best scale for their goals, there are a number of additional scenarios where the database may prove to be a useful resource. For example, when developing a new scale, researchers need existing validated scales to support the validation process (a critical step in the scale development process (Cronbach and Meehl, 1955; Messick, 1989; Raykov and Marcoulides, 2011)). In these cases, the database can be a helpful resource. This is because the scales are organized by construct or research domain and ranked within these categories by guideline rating, which simplifies the search for measures that can be used to assess scale validity. For example, if a researcher is developing a scale to assess the perception of rapport with a robot, they may want to use the Human-Robot Interaction Evaluation Scale (HRIES) (Spatola et al., 2021) or the Connection-Coordination Rapport Scale (CCR) (Lin et al., 2025) to assess their scale’s convergent validity with the current best scales within that construct according to our guideline. Similarly, if the same researcher is searching for a measure that can be used to assess the discriminant validity of their scale (i.e., the extent to which the scale differs from other unrelated constructs (Raykov and Marcoulides, 2011)), they can easily scan the list of constructs to find a scale that is highly rated per the guideline and unrelated to rapport (e.g., perceived danger scale (Molan et al., 2025)).

The database may also be useful to those conducting literature reviews or meta-analyses on specific constructs commonly investigated in HRI. The database streamlines the search for the most commonly used scales for a specific construct, making it easier to compare how construct definitions vary across scales and how different scales measure the construct in similar or distinct ways. These types of analyses may also lead to the development of new scales where gaps in the literature are identified. Across all of these applications, the database can be used as a valuable research tool saving HRI researchers time and resources and simplifying the process of choosing the best scale for their projects. For those HRI researchers interested in incorporating scales into their research, we recommend adding the HRI scale database as a tool in your toolbox. Our aim is to simplify the process of finding and evaluating scales for research, so that researchers can be confident that their measures are as strong as they can be. It is our hope that the database is a useful tool for HRI researchers at all levels and improves the quality of research in this domain and in the field in general.

## Data Availability

The original contributions presented in the study are included in the article/supplementary material, further inquiries can be directed to the corresponding author. The database is publicly available and can be accessed online at https://hriscaledatabase.psychology.gmu.edu/.

## Footnotes

1 An additional potential benefit is that the database and guideline ratings will also help push the field away from choosing scales based solely on frequency of use, which is not always indicative of measurement quality.

2 The guideline was developed by the authors prior to the creation of the database and independently of the scale selection process. Its development was intended to provide HRI researchers with a general framework for evaluating the psychometric quality and reporting practices of HRI scales, rather than to assess any specific set of measures. The database was subsequently created as an application of this framework. In applying the guideline, we used a deliberately conservative and inclusive approach which focused on whether any of the minimum recommended psychometric criteria were reported in the original scale development and validation publications.

3 It is important to note that the guideline evaluates the extent to which recommended psychometric development and validation practices were reported in the original publications, rather than directly measuring the underlying quality of the scales themselves. Consequently, lower scores for older scales may partially reflect historical differences in reporting standards and publication norms rather than inherently poorer scale development practices. We thank an anonymous reviewer for raising this point.

4 We note that a direct implication of this result is that more recently developed measures will be less frequently cited. This may temporarily complicate comparisons with prior work which relies on more established instruments. However, this should not discourage the adoption of newer scales, since as they are increasingly used, their cumulative evidence base will grow allowing for comparability with more accurate measures. As a result, the use of these measures will ultimately strengthen theory and measurement in the field. Although the continued use of legacy measures is still common, we hope that the guideline (Saad et al., 2025) and database presented here supports researchers in confidently selecting and justifying the use of higher-quality (and potentially less frequently cited) scales in future work.
